# Interleukin-6 Deficiency Does Not Affect Motor Neuron Disease Caused by Superoxide Dismutase 1 Mutation

**DOI:** 10.1371/journal.pone.0153399

**Published:** 2016-04-12

**Authors:** Yongmei Han, Barry Ripley, Satoshi Serada, Tetsuji Naka, Minoru Fujimoto

**Affiliations:** 1 Graduate School of Frontier Biosciences, Osaka University, 1–3 Yamadaoka, Suita, Osaka 565–0871 Japan; 2 Laboratory of Immune Signal, National Institute of Biomedical Innovation, Health and Nutrition, Osaka, Japan; 3 Laboratory of Immune Regulation, IFReC Research Building, Osaka University 3–1 Yamadaoka, Suita, Osaka 565–0871, Japan; University of Nebraska Medical center, UNITED STATES

## Abstract

**Background & Aim:**

Amyotrophic Lateral Sclerosis (ALS) is an adult-onset, progressive, motor neuron degenerative disease. Recent evidence indicates that inflammation is associated with many neurodegenerative diseases including ALS. Previously, abnormal levels of inflammatory cytokines including IL-1β, IL-6 and TNF-α were described in ALS patients and/or in mouse ALS models. In addition, one study showed that blocking IL-1β could slow down progression of ALS-like symptoms in mice. In this study, we examined a role for IL-6 in ALS, using an animal model for familial ALS.

**Methods:**

Mice with mutant SOD1 (G93A) transgene, a model for familial ALS, were used in this study. The expression of the major inflammatory cytokines, IL-6, IL-1β and TNF-α, in spinal cords of these SOD1 transgenic (TG) mice were assessed by real time PCR. Mice were then crossed with IL-6(-/-) mice to generate SOD1TG/IL-6(-/-) mice. SOD1 TG/IL-6(-/-) mice (n = 17) were compared with SOD1 TG/IL-6(+/-) mice (n = 18), SOD1 TG/IL-6(+/+) mice (n = 11), WT mice (n = 15), IL-6(+/-) mice (n = 5) and IL-6(-/-) mice (n = 8), with respect to neurological disease severity score, body weight and the survival. We also histologically compared the motor neuron loss in lumber spinal cords and the atrophy of hamstring muscles between these mouse groups.

**Results:**

Levels of IL-6, IL-1β and TNF-α in spinal cords of SOD1 TG mice was increased compared to WT mice. However, SOD1 TG/IL-6(-/-) mice exhibited weight loss, deterioration in motor function and shortened lifespan (167.55 ± 11.52 days), similarly to SOD1 TG /IL-6(+/+) mice (164.31±12.16 days). Motor neuron numbers and IL-1β and TNF-α levels in spinal cords were not significantly different in SOD1 TG /IL-6(-/-) mice and SOD1 TG /IL-6 (+/+) mice.

**Conclusion:**

These results provide compelling preclinical evidence indicating that IL-6 does not directly contribute to motor neuron disease caused by SOD1 mutations.

## Introduction

Amyotrophic lateral sclerosis (ALS) is a chronic fatal neurodegenerative disease characterized by progressive motor paralysis due to degeneration of upper and lower motor neurons in the brain and spinal cord. Generally, death results from respiratory failure due to paralysis of the respiratory muscles. Although 90% of all ALS cases are sporadic, there are also genetic causes, such as mutations in superoxide dismutase 1 (SOD1) [1,2], which are clinically and pathologically similar to sporadic ones. Transgenic mouse models overexpressing mutant human SOD1 also manifest symptoms of ALS [3]. These mice are widely used to examine mechanisms of the disease and screen for therapeutic targets.

Currently there is no curative treatment for ALS. Only riluzole increases the life span of the patients with an average of 2–3 months [4]. As a consequence, a detailed understanding of the disease mechanism as well as the search for new and better treatment strategies is a top priority for ALS. Several mechanisms have been proposed to explain motor neuron death in ALS, including glutamate-induced excitotoxicity, ER stress, proteasome inhibition, mitochondrial dysfunction and O_2_^-^ production [5]. However, the underlying mechanisms are still poorly understood.

Accumulating evidence indicates that the immune system plays a role in the development of ALS [6] in which microglia overexpressing mutant SOD1 induce pro-inflammatory cytokines, such as TNFα and IL-1β [7]. Consistently, abnormal levels of inflammatory cytokines including TNF-α and IL-1β were described in ALS patients and/or ALS model mice [8]. Moreover, one study showed that blocking IL-1β could slow down progression of ALS-like symptoms in mice [9].

IL-6 is considered to be a pro-inflammatory cytokine that plays a key role in immune responses [10]. Like other pro-inflammatory cytokines such as TNFα and IL-1β, IL-6 is an important therapeutic target of immune disorders, and anti-IL-6 receptor antibody, tocilizumab, has been developed as a therapy in human diseases, including rheumatoid arthritis (RA) and Castleman’s disease [11]. Moreover, a considerable number of case reports and pilot studies have indicated the beneficial effects of tocilizumab on other autoimmune and chronic inflammatory diseases [12]. Interestingly, as summarized in a recent meta-analysis [13], several studies indicated that IL-6 is elevated in the spinal cord of the mouse ALS model [14,15]. In addition, a previous report showed that IL-6 is increased in the cerebrospinal fluid of ALS patients [16]. Furthermore, recent preliminary studies by Fiala et al. have shown that tocilizumab treatment can modulate the inflammatory gene signature in peripheral blood cells of sporadic ALS patients and may possibly decelerate disease progression [17,18]. Given that the involvement of IL-6 in the pathophysiology of ALS models remains unanswered, we have assessed whether IL-6 deficiency influences the onset and progression of motor neuron disease in a murine model of ALS.

## Materials and Methods

### Animals

SOD1 (G93A) transgenic mice, which express the human SOD1 gene containing a G93A mutation on a C57BL/6 background (B6.Cg-Tg (SOD1-G93A) 1Gur/J; stock No. 004435) were obtained from Jackson Laboratory. The survival of SOD1 TG mice is known to vary depending on the genetic background and housing condition [19] and median lifespan of SOD1 TG mice in this study was close to or slightly longer than that reported by Jackson Laboratory (157.1 ± 9.3 days). SOD1 TG IL-6(-/-) mice was generated by mating SOD1 TG males with IL-6(-/-) females [20]. All mice were maintained under specific pathogen-free conditions at the National Institute of Biomedical Innovation, Health and Nutrition (NIBIOHN). Animal experiments were approved by the Animal Care and Use Committee of NIBIOHN (DS25-34) and were carried out according to the institutional ethical guidelines for animal experiments of NIBIOHN (Osaka, Japan).

### Experimental groups

SOD1 TG/IL-6(-/-) mice (n = 17) were compared with SOD1 TG /IL-6(+/-) mice (n = 18), SOD1 TG /IL-6(+/+) mice (n = 11), WT mice (n = 15) IL-6(+/-) mice (n = 5) and IL-6(-/-) mice (n = 8) from 8 weeks of age, with respect to neurological disease severity score, body weight and the survival.

### RNA Isolation and Real-time Quantitative Polymerase Chain Reaction (PCR)

Total RNA was extracted individually from the whole spinal cord of SOD1 TG/IL-6(-/-) mice, SOD1 TG /IL-6(+/+) mice, IL-6(-/-) mice and WT mice at 150 to 160 days of age. Each group or condition had 6–8 mice. Total RNA was isolated with Sepasol-RNA1 Super G (Nacalai, Kyoto, Japan) and was cleaned up with RNeasy mini spin columns (Qiagen, Valencia, GA) following the manufacturer’s instructions. Total RNA (500 ng) was reverse-transcribed into first-strand complementary DNA (cDNA) (QuantiTect Reverse Transcription Kit, Qiagen). The primers used for real-time PCR were as follows: IL-6, sense 5' -tggctaaggaccaagaccatccaa-3', antisense 5'- aacgcactaggtttgccgagtaga -3';IL-1β, sense 5'- aaacggtttgtcttcaac-3', antisense 5'- atggtgaagtcaattatgtc-3';TNF-α,sense 5'-ctgtgaagggaatgggtgtt -3',antisense 5'-cccagcatcttggtttctg-3'; Each reaction was performed in triplicate with FastStart universal SYBR Green Master(roche, Mannheim, Germany) and was analyzed by the 7900HT real time PCR system (Applied Biosystems, Foster City, CA).

### Monitoring Disease Progression

Neurological scores for both hind legs were assessed twice a week for each mouse from 8 weeks of age. The neurological score employed a scale of 0 to 4 that was developed by observation at ALS TDI [21,22]. Criteria used to assign each score level were:

1. Full extension of hind legs away from lateral midline when mouse is suspended by its tail, and mouse can hold this for 2 seconds, suspended 2–3 times.
2. Collapse or partial collapse of leg extension towards lateral midline (weakness) or trembling of hind legs during tail suspension.
3. Toes curl under at least twice during walking of 12 inches, or any part of foot is dragging along cage bottom/table.
4. Rigid paralysis or minimal joint movement, foot not being used for forward motion.
5. Mouse cannot right itself within 30 seconds from either side.

In this study, the onset was determined by the development of overt paralytic symptoms (score criteria 2), as reported previously [21]. Based on the pre-study observation of the clinical score, two time points, the early stage (80–100 days of age) at which SOD1 TG mice exhibited mild symptoms (score criteria 1) and the end stage (155–175 days of age) during which mice exhibited rapid deterioration of the disease (score criteria from 2 to 4), were chosen for detailed analyses. To treat mice humanely, food and water (Nutra-Gel) was provided at the bottom of the cage, when mice dragged their hind legs due to the disease progression. Mice that attained score criteria 4 were euthanized and excluded from further experiments.

### Survival

To determine survival, mice were monitored every day and, for humane reasons, were euthanized before their actual death if mice could not right themselves within 30 seconds from either side (score criteria 4). Isoflurane anesthesia followed by cervical dislocation was used to sacrifice mice.

### Body weight

Body weight measurements were recorded for each animal beginning at 57 days of age.

### Spinal cord Nissl staining, motor neuron count and muscle Hematoxylin-Eosin (HE) staining

SOD1 TG/IL-6(-/-) mice, SOD1 TG /IL-6(+/+) mice and WT mice were anesthetized and sacrificed at the early stage and the end stage. Mice were perfused with cold phosphate-buffered saline (PBS). Lumbar spinal cords (n = 4) were dissected carefully and tissues were immediately fixed in 10% formalin neutral buffer solution and embedded in paraffin. Blocked spinal cord was cut into 5 μm of cross-sections. Slices of spinal cord (5 μm-thick cross-sections) were stained with Cresyl violet solution (Nissl staining; MUTO pure Chemicals, Tokyo, Japan) and were observed by Leica DM5500 microscope (n = 4–5 animals per group). For quantitative analysis of motor neurons in the spinal cord, sections with an interval of 1mm tissue were chosen for evaluation to avoid double-counting of the same neurons. The slides were visualized with a microscope at 100-fold magnifications and one ventral horn of one tissue section was analyzed per slide. Each Nissl stained neuron with a distinct nucleolus and a darkly stained cytoplasm was captured manually using BZ-II analyzer software to determine cross-sectional area. As reported previously [23], α- and γ-motor neurons were defined as having cross-sectional area ranging from 250 to 1100 μm^2^ and from 100 to 250 μm^2^, respectively. For muscle HE staining, right hamstring muscle tissues were immediately fixed in 10% formalin neutral buffer solution and embedded in paraffin. Paraffin sections (2.5 μm) were stained with hematoxylin and eosin (n = 5 per group). The slides were visualized with a microscope (Leica) at 400-fold magnifications.

### Statistical analyses

The student’s two-tailed t-test (Microsoft Excel software, Redmond, WA) was used for comparison between two groups. When analysis included more than two groups, we used one-way analysis of variance followed by Tukey-Kramer post-hoc test. Alternatively, when the data (> two groups) did not follow a normal distribution or did not have similar variances, data were analyzed by Kruskal-Wallis test followed by Scheffe’s multiple comparison. Time-to-onset and survival rates were analyzed by the Kaplan-Meier method and were compared by the log-rank test using Ekuseru-Toukei 2012. P-values less than 0.05 were considered statistically significant.

## Results

### IL-6 expression is increased in SOD1 TG mice

**Fig 1:**
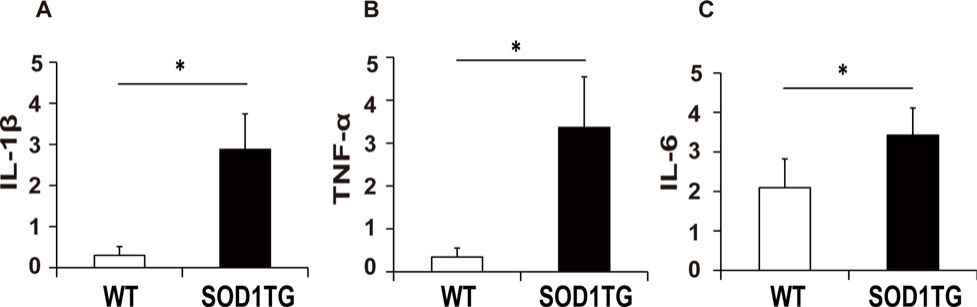
Quantitative Real Time PCR of IL-1β, TNFα and IL-6 in spinal cord of SOD1 TG mice and WT mice. Messenger RNA obtained from spinal cord of SOD1 TG mice (n = 4) and WT mice (n = 5) was subjected to real-time PCR analysis of IL-1β **(A)**, TNF-α **(B)** and IL-6 **(C)** expression (*p≤0.05 by t-test).

To assess the involvement of inflammatory cytokines in the pathogenesis of ALS model mice, we first compared the expression of IL-1β, TNF-α and IL-6 in the spinal cord of SOD1 TG mice and wild-type mice by real-time quantitative PCR. As reported previously [8], levels of IL-1β and TNF-α were elevated in SOD1 TG mice compared to WT mice. (Fig 1A and 1B). In addition, we found that IL-6 levels were also significantly higher in SOD1 TG mice compared to WT mice (Fig 1C), suggesting that IL-6 may be a therapeutic target of ALS.

### IL-6 deficiency does not influence disease progression in ALS mice

To assess the contribution of IL-6 in ALS pathogenesis, we generated SOD1 (G93A) transgenic mice in the context of IL-6 gene knock-out.

**Fig 2:**
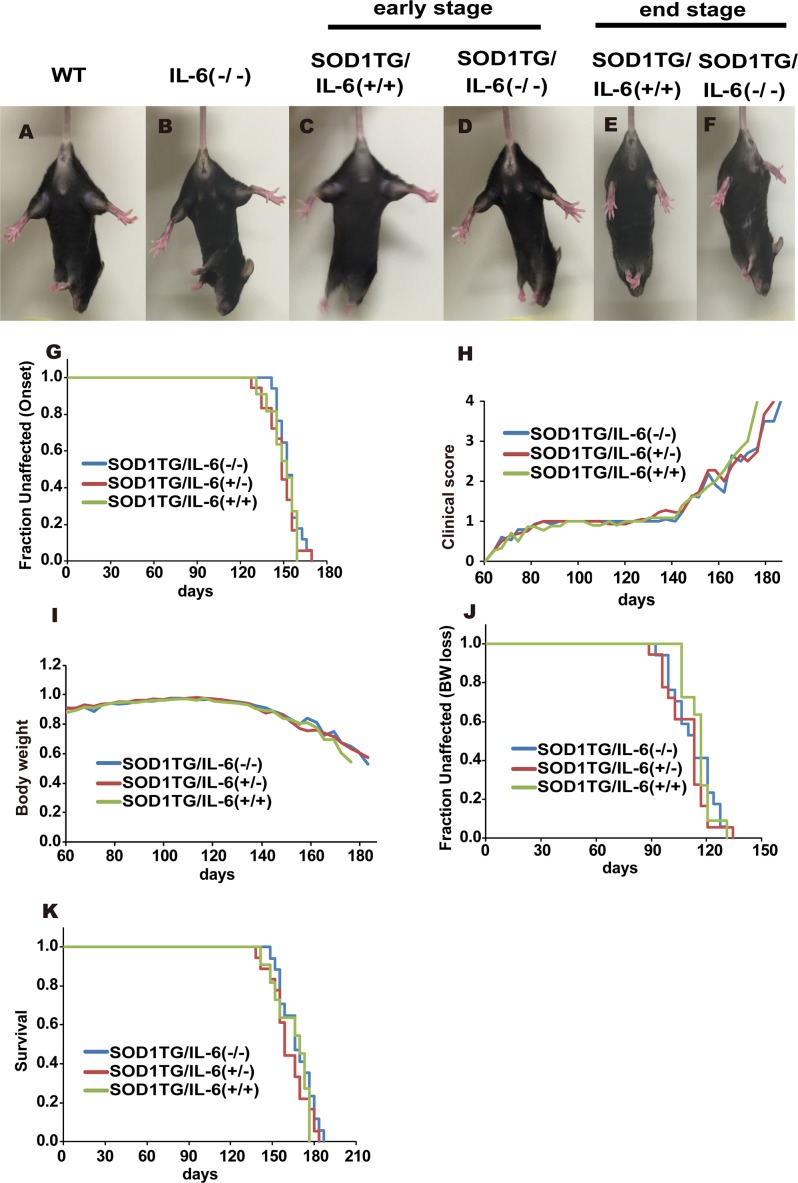
Relative changes in body weight, clinical score and survival in ALS mice. **(A-F)** Hind limb extension of WT mouse, IL-6(-/-) mouse (160–180 days of age), SOD1 TG/IL-6(+/+) mice and SOD1 TG/IL-6(-/-) mice (the early stage and the end stage), when suspended by their tail. **(G)** Kaplan-Meier time–to-failure plot for onset of symptomatic neurological disease in ALS mice. Neurological symptoms of SOD1 TG/IL-6(-/-) (n = 17), SOD1 TG/IL-6(+/-) (n = 18) and SOD1 TG/IL-6(+/+) (n = 11) were monitored. The age at which mice attain a neurological severity score 2 is taken to be definitive onset of symptomatic neurological disease (p = 0.597 by log rank test for onset of symptomatic neurological disease). **(H)** The clinical score of SOD1 TG/IL-6(-/-) mice in comparison with those of SOD1 TG/IL-6(+/-) mice and SOD1 TG/IL-6(+/+) mice. Score criteria (severity from 0 to 4) are shown in materials and methods. SOD1 TG/IL-6 (-/-) (n = 17), SOD1 TG/IL-6(+/-) (n = 18), SOD1 TG /IL-6(+/+) (n = 11), IL-6(-/-) (n = 8), WT (n = 15), IL-6(+/-) (n = 5). WT, IL-6(-/-) or IL-6(+/-) mice showed no disease symptoms. **(I)** Relative changes in body weight of SOD1 TG/IL-6(-/-) mice (n = 17), SOD1 TG /IL-6(+/-) mice (n = 18) and SOD1 TG /IL-6(+/+) mice (n = 11). Peak body weight of each mouse was calculated as 1. (**J**) Kaplan-Meier time–to-failure plot for onset of body weight loss of SOD1 TG/IL-6(-/-), SOD1 TG/IL-6(+/-) and SOD1 TG/IL-6(+/+) mice. The age at which mice attained a peak body weight was analyzed (p = 0.595 by log rank test). (**K**) Kaplan-Meier time–to-failure plot for survival of SOD1 TG/IL-6(-/-), SOD1 TG/IL-6(+/-) and SOD1 TG/IL-6(+/+) mice (p = 0.430 by log rank test). The survival of SOD1 TG/IL-6(-/-) (n = 17), SOD1 TG/IL-6(+/-) (n = 18) and SOD1 TG/IL-6(+/+) (n = 11) was monitored. The mean (± SD) life spans were; SOD1 TG/IL-6(-/-) 167.55 ± 11.52 days, SOD1 TG/IL-6(+/-) 161.82 ± 12.69 days, TG/IL-6(+/+) 164.31 ± 12.16 days.

The effect of IL-6 deficiency on the severity of motor neuron disease was examined using cohorts of SOD1 TG/IL-6(+/+) mice and SOD1 TG/IL-6(-/-) mice. When suspended by the tail, both SOD1 TG/IL-6(-/-) mice and SOD1 TG/IL-6(+/+) mice at the early stage (80–100 days of age) exhibited mild hind limb tremor (data not shown) but could extend their limbs similarly to non-TG mice (Fig 2A, 2B, 2C and 2D). However, at the end stage (155–175 days of age), hind limb extension was impaired in both SOD1 TG/IL-6(-/-) mice and SOD1 TG/IL-6(+/+) mice (Fig 2E and 2F), suggesting the motor neuron disease progressed similarly in these mutant mice. Indeed, as shown in (Fig 2G), the lack or reduction of IL-6 did not delay the onset of the definitive paralytic symptoms (as defined by attaining the score 2, as reported previously [21]) in SOD1 TG mice (p = 0.597 by log rank test). Mean (± SD) days at onset (score 2) for SOD1 TG/IL-6(-/-), SOD1 TG/IL-6(+/-) and SOD1 TG/IL-6(+/+) mice were 153.34 ± 7.50, 148.59 ± 10.23 and 149.67 ± 8.86 days, respectively and the clinical scores of these mice increased similarly thereafter (Fig 2H). In addition, their body weight decreased comparably as they aged (Fig 2I), peaking at 112.58 ± 11.71, 109.51 ± 11.44 and 115.63 ± 7.39 days for SOD1 TG/IL-6(-/-), SOD1 TG/IL-6(+/-) and SOD1 TG/IL-6(+/+) mice, respectively (p = 0.595 by log rank test, Fig 2J). Furthermore, SOD1 TG/IL-6(-/-) mice, SOD1 TG/IL-6(+/-) mice and SOD1 TG/IL-6(+/+) mice displayed comparable mean lifespans of 167.55 ± 11.52, 161.82 ± 12.69 and 164.31 ± 12.16 days, respectively, and all of them died before reaching 190 days old (p = 0.430 by log rank test, Fig 2K). These results indicate that the lack or reduction of IL-6 did not influence onset, disease progression or mortality in mutant SOD1 transgenic mice.

### IL-6 deficiency does not affect motor neuron loss and muscle atrophy in ALS mice

**Fig 3:**
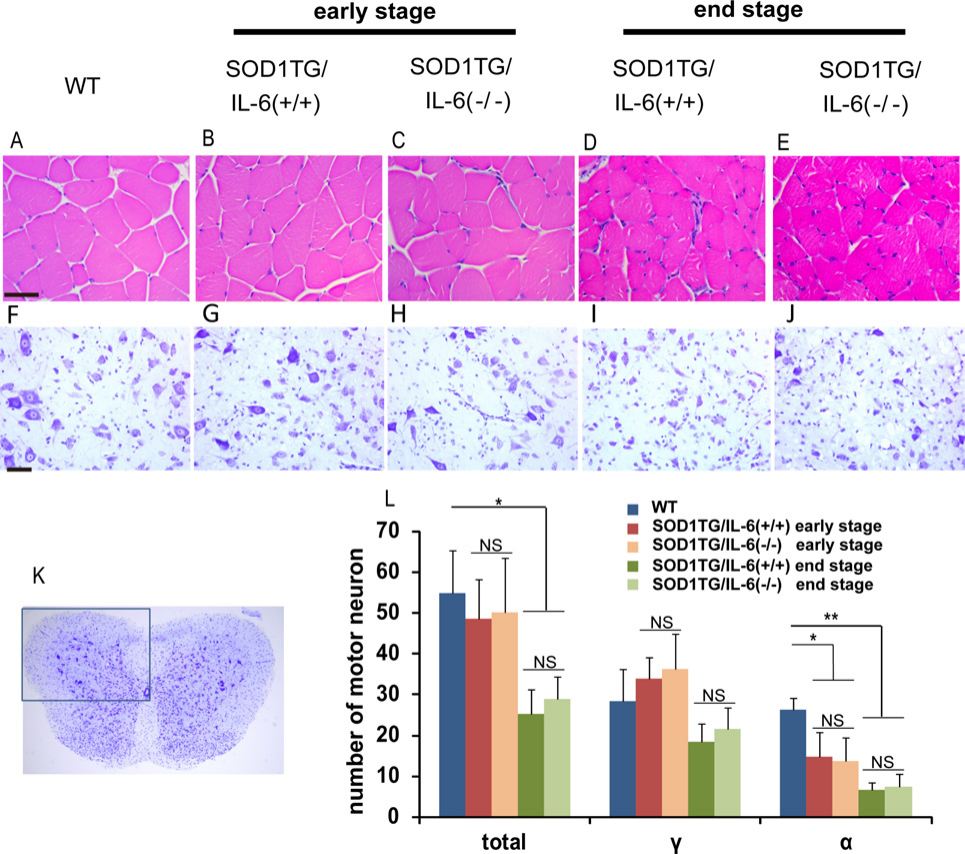
Histological analysis of motor neuron loss and muscle atrophy in ALS mice. (A, B, C, D, E) HE staining of hamstring muscles from the early stage and the end stage of ALS mice and control mice with indicated genotype. Scale bars: 50 μm. (F, G, H, I, J) Lumber spinal cords from mice with indicated genotypes at the early and the end stage were stained with Cresyl violet solution (Nissl staining). Scale bars: 50μm. (K) A representative image of Nissl-stained mouse spinal cord. Boxed area is a region defined as the anterior horn for motor neuron counting. (L) Quantitative cell counts of motor neurons in the anterior horn section. Spinal cord sections from mice with indicated genotype at the early and the end stage were stained with Cresyl violet solution and motor neurons were counted (*p<0.05 and **p<0.01 by Tukey Kramer post-hoc test).

Histological analysis of hamstring muscles revealed that muscle fiber atrophy became detectable in SOD1 TG/IL-6(+/+) mice at the early stage (Fig 3B) and progressed thereafter at the end stage (Fig 3D). However, no differences were observed in muscle atrophy between the SOD1 TG/IL-6(-/-) mice and SOD1 TG/IL-6(+/+) mice at both early and end stage (Fig 3C and 3E).

To determine whether IL-6 deficiency affects spinal cord pathology, we performed Nissl staining of lumber spinal cords from wild type mice, SOD1 TG/IL-6(-/-) mice and SOD1 TG/IL-6(+/+) mice at the early stage and the end stage (Fig 3F, 3G, 3H, 3I and 3J). In SOD1 TG/IL-6(+/+) mice at the early stage of the disease, there was a slight loss of motor neurons (Fig 3G), which became evident at the end stage (Fig 3I). Similar motor neuron loss was observed in SOD1 TG/IL-6(-/-) mice at the early and the end stage (Fig 3H and 3J). To confirm this result quantitatively, motor neurons in the anterior horn (boxed area; Fig 3K) were counted as defined in Materials and methods. The numbers of total motor neurons were significantly reduced at the end stage both in SOD1 TG/IL-6(+/+) mice and in SOD1 TG/IL-6(-/-) mice. In particular, while the numbers of γ motor neurons were not significantly altered, the numbers of α motor neurons that generate force to move the skeleton [24] were significantly reduced at the early stage and was further decreased at the end stage both in SOD1 TG/IL-6(+/+) mice and in SOD1 TG/IL-6(-/-) mice (Fig 3L). Because there was no significant difference in motor neuron numbers between SOD1 TG/IL-6(+/+) mice and SOD1 TG/IL-6(-/-) mice, it is likely that IL-6 deficiency does not affect motor neuron loss in this model.

### IL-6 deficiency does not affect inflammatory cytokine levels in spinal cord of ALS mice

**Fig 4:**
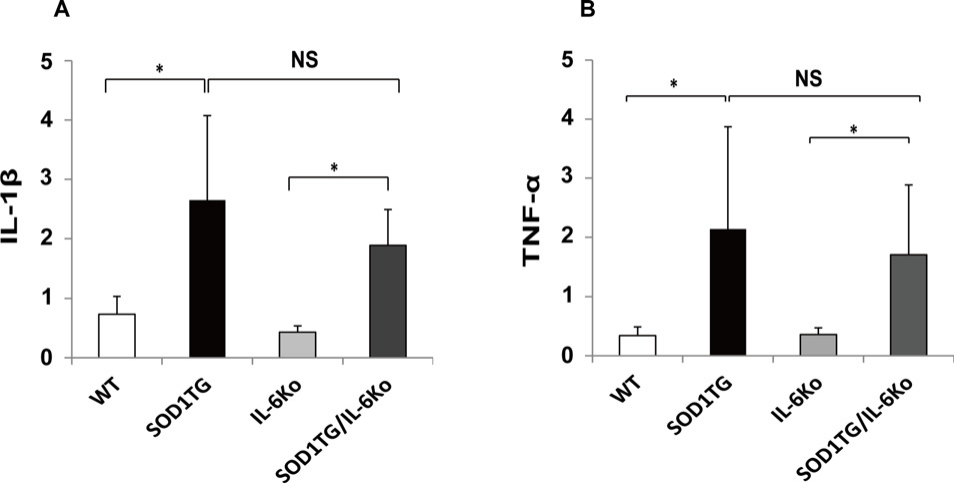
Quantitative Real Time PCR of IL-1β and TNF-α in spinal cords of SOD1 TG/IL-6(-/-) mice and control mice. Messenger RNA obtained from spinal cord of SOD1 TG mice (n = 7), SOD1 TG/IL-6(-/-) mice (n = 7), IL-6 knockout mice (n = 6) and WT mice (n = 8) was subjected to real-time PCR analysis of IL-1β **(A)**, TNF-α **(B)** expression (* p≤0.05 by Scheffe’s multiple comparison).

IL-6 has various stimulatory effects on immune cells and plays a key role in propagating inflammation in autoimmune diseases such as RA. To investigate if IL6-deficiency influences the inflammatory response in the spinal cord of ALS mice, levels of other pro-inflammatory cytokines, IL-1β and TNF-α were examined by real-time quantitative PCR. As a result, IL-1β and TNF-α levels were increased in SOD1 TG/IL-6(-/-) mice and SOD1 TG/IL-6(+/+) mice compared to WT mice and IL-6 knockout mice (Fig 4A and 4B) and were not significantly different between SOD1 TG/IL-6(-/-) mice and SOD1 TG/IL-6(+/+) mice. These data indicate that IL-6 is not necessary for the induction of IL-1β and TNF-α in spinal cord, and suggest that IL-6 deficiency does not affect the neural inflammatory response caused by SOD1 mutation.

## Discussion

Recently, a number of biologic agents targeting inflammatory cytokines, such as IL-6, IL-1β and TNF-α have been developed and used as therapeutics for RA and other immune disorders. These inflammatory cytokines are expressed at the lesion sites in mice and humans, and blocking one of these molecules has proven to be highly effective in the treatment [25,26,27,28,29]. Given the reported involvement of the immune system and inflammatory cytokines in neurodegenerative diseases including ALS [8,30], similar strategy targeting cytokines would be efficacious in treating these intractable diseases.

In this study, we found that the level of IL-6, as well as those of IL-1β and TNF-α, was significantly elevated in the spinal cord of SOD1 TG mice (Fig 1A, 1B and 1C). Although there are some inconsistencies in the literature, this finding is supported by a recent meta-analysis of SOD1 TG mouse studies [13]. However, unexpectedly, we report here that disruption of the IL-6 gene in mice failed to influence onset, severity, or progression of disease caused by SOD1 mutation. These results suggest that IL-6 is not a crucial contributor to motor neuron degeneration in this murine model.

IL-6 is a multifunctional cytokine that functions not only in immune cells but also in other cell types including neurons [31]. Previous studies on neurological disease models indicate that IL-6 can accelerate or decelerate neuronal regeneration in a context-dependent manner [31]. In this study, however, motor neuron loss in the spinal cord was similar between SOD1 TG/IL-6(-/-) mice and in SOD1 TG/IL-6(+/+) mice. This result suggests that IL-6 is not necessary to regulate motor neuron survival or death in this model.

Recent pilot study by Fiala et al has shown that IL-6 blockade by tocilizumab in sporadic ALS patients in vivo could modulate the inflammatory gene signature in their peripheral blood cells [17]. However, our study on a murine model of familial ALS indicated that the absence of IL-6 did not influence expression levels of IL-1β and TNF-α in the spinal cord of SOD1 TG mice (Fig 4). Thus, our results suggest that IL-6 plays a minor role in the neural inflammation caused by mutant SOD1. It should be noted, however, that the clinical study of IL-6 blockade is conducted on patients with sporadic ALS, but not with familial ALS. Future clinical studies would be important to determine whether IL-6 blockade in human ALS can modulate neuronal inflammatory gene signature of these patients.

We have shown previously that blocking IL-6 action can ameliorate inflammation in a variety of murine autoimmune diseases, mainly by modulating T helper cell differentiation [20,32,33,34]. These observations indicate that IL-6 blockade is an effective treatment for the immune disorders where the disease is driven by the adaptive immune system. However, according to the recent studies using SOD1 TG mice lacking lymphocytes, the adaptive immune system is not pathogenic in motor neuron disease, but rather may be protective against the disease progression [35,36,37]. Moreover, surprisingly, others have reported that boosting autoimmune T cells in ALS mice by immunizing myelin-derived autoantigen did not accelerate disease but rather attenuated disease progression [38,39]. Furthermore, the absence of TNF-α failed to ameliorate motor neuron disease in SOD1 TG mice [40]. These findings may collectively indicate that there is no benefit in suppressing adaptive immunity and/or autoimmune responses in ALS. Thus, unlike autoimmune diseases such as RA, IL-6 and TNF-α may not be ideal therapeutic targets of ALS and the elevation of these two cytokines may be due to a secondary event of neuroinflammation rather than a cause of disease.

It should be noted, however, that immune responses in ALS are complex and are likely to have dual roles [41] which are presumably regulated by the balance between protective and pathogenic cytokines [13] and/or cell types [42]. Indeed, lines of evidence still indicate that the immune system contributes to the progression of ALS. Several studies of ALS models concluded that microglial cells, the macrophages in the central nerve system, that express mutant SOD1 play a key role in rapid progression of the symptoms [5]. In addition, one study showed that SOD1-induced IL-1β accelerates ALS pathogenesis [9]. The contribution of IL-1β signaling in ALS pathogenesis was further exemplified by the findings that gene deletion of either IL-1β or caspase-1 [9] or intracerebroventricular administration of a caspase inhibitor [43] could slow disease progression in SOD1 TG mice. Given the close linkage of IL-1β to innate signaling pathways, aberrant activation of innate immunity but not of adaptive immunity might drive the disease progression in ALS. Recently, a new class of immune-mediated diseases was proposed and named as autoinflammatory diseases. The mechanism of these diseases is different from that of autoimmunity and the defect is located in innate immunity, not in adaptive immunity [44]. Interestingly, while IL-1 blockade was considered less superior than TNF-α blockade in treating RA and other autoimmune diseases, it was remarkably effective in treating autoinflammatory diseases [43]. In this context, inflammation that accelerates ALS pathology may be driven mainly by innate immunity and may involve a mechanism resembling autoinflammatory diseases. Clearly, further studies are required to clarify this question.

In conclusion, our data suggest that IL-6 is not a key factor in motor neuron disease caused by SOD1 mutation. Taken together with previous studies by others, current biologic agents including IL-6 blockade and TNF inhibitors, highly efficacious in treating autoimmune diseases, may not work well for treating ALS.

## Abbreviation

ALS: Amyotrophic lateral sclerosis
WT: wild type
SOD: superoxide dismutase
TG: transgenic
IL-6: interleukin-6
IL-1: interleukin-1
TNF-α: tumor necrosis factor-alpha
NF-kB: nuclear factor-kappa B
PBS: phosphate buffered saline
